# Cognitive Impairment Early After Initiating Maintenance Hemodialysis: A Cross Sectional Study

**DOI:** 10.3389/fneur.2022.719208

**Published:** 2022-03-15

**Authors:** Melissa Schorr, Mariah Zalitach, Cindy House, Janice Gomes, Conor J. Wild, Fabio R. Salerno, Christopher McIntyre

**Affiliations:** ^1^Division of Nephrology, Department of Medicine, London Health Sciences Centre, London, ON, Canada; ^2^Department of Health Research Methods, Evidence and Impact, McMaster University, Hamilton, ON, Canada; ^3^The Lilibeth Caberto Kidney Clinical Research Unit, London Health Sciences Centre, London, ON, Canada; ^4^Department of Pathology and Laboratory Medicine, University of Western Ontario, London, ON, Canada; ^5^Brain and Mind Institute, University of Western Ontario, London, ON, Canada; ^6^Department of Medical Biophysics, Western University, London, ON, Canada

**Keywords:** hemodialysis, cognitive impairment, Cambridge Brain Sciences, memory, verbal, reasoning, cognitive domains

## Abstract

**Background:**

Abnormalities in cognitive function are almost universal in patients receiving hemodialysis (HD) and are associated with worse quality of life, impaired decision making, increased healthcare utilization and mortality. While cognitive impairment in the HD population is increasingly recognized, it is unclear how quickly it develops after starting HD.

**Methods:**

This was a cross-sectional study of a cohort of low dialysis vintage HD patients (<12 months). We used the validated Cambridge Brain Science (CBS) battery of web-based tests to evaluate cognition compared to age- and sex matched controls across three cognitive domains: verbal processing, reasoning and short-term memory.

**Results:**

Forty-nine HD patients were included in this study; 43 completed the full battery of tests. The average scores for HD patients were consistently below the age and sex-matched controls. Fifty-five percent of HD patients had cognitive impairment in verbal skills, 43% in reasoning and 18% in short-term memory.

**Conclusions:**

There is a high prevalence of CI evident early after starting HD, with the largest deficits seen in reasoning and verbal processing. These deficits may be attributable to the HD treatment itself. Further studies are needed to characterize the natural history of CI in this patient population and to test interventions aimed at preventing or slowing its progression.

## Introduction

Neurological disorders including ischaemic brain injury, cognitive impairment (CI) and dementia are becoming increasingly recognized in hemodialysis (HD) patients (1–3). Abnormalities in cognitive function are almost universal in this patient population (4, 5). Mild CI and dementia—in particular, vascular dementia—are significantly more prevalent in HD patients than the general population (6–9).

### Etiology of CI

There are a number of factors that may contribute to CI and dementia in CKD and ESRD including accumulation of uremic toxins, cerebral vascular dysfunction, chronic inflammation, anemia and white matter injury in addition to established risk factors such as advanced age and depression (10–13). Compared to patients with chronic kidney disease (CKD) or undergoing peritoneal dialysis (PD), CI appears to be more common in HD patients, suggesting unique contributing factors in these patients (14–16).

### Relevance of CI

Impaired cognitive function is associated with depression (17), non-adherence (18) and a worse quality of life (19). It may result in poor self-care, impaired ability to make informed decisions and increased healthcare utilization (19–21). CI has also been associated with increased mortality in HD patients (1, 22, 23).

### Testing for CI

In an American study by Drew et al., the performance of established screening tests for CI was evaluated in the HD population. The best performing test was the Montreal Cognitive Assessment (MoCA), a 30-question test administered by trained healthcare professionals (https://www.mocatest.org/) (24). This test predicted CI with high sensitivity and moderate specificity (a score of ≤ 21 had a sensitivity of 86% and specificity of 55% for severe impairment), however, it requires trained healthcare personnel to administer it (24).

An alternate tool is the Cambridge Brain Sciences (CBS) neurocognitive test which is a web-based battery of 12 tests that comprehensively evaluates cognition. The details of each test have been well-described elsewhere (25, 26). This tool can be self-administered with automated scoring and obviates the need for trained personnel. There is a well-established database of healthy controls available for matching, and the tool has been widely validated and used in clinical research (26–28). It has been used internationally among a culturally diverse populations including in individuals with structural brain abnormalities as well as neurodegenerative diseases (25, 26, 28–30).

To the best of our knowledge, however, the CBS has not been previously used in the HD patient population.

### Objectives

The objectives of this cross-sectional study are (1) to use the CBS tests to measure CI within the first year of HD and (2) to describe the patterns of CI early after starting HD. The CBS battery to patients who have been on maintenance hemodialysis for <12 months and compare their scores to a database of age- and gender-matched controls.

## Materials and Methods

We conducted a cross sectional study of new start hemodialysis patients at eight hemodialysis centers in Southern Ontario associated with London Health Sciences Center. The study was approved by our local research ethics board (Western REB, study identification number 111721).

We included adult patients (≥18 years of age) who had been on maintenance hemodialysis for a minimum of 30 days but <12 months. Patients were excluded if they had a pre-existing diagnosis of dementia, new or pre-existing diagnosis of neurological disease known to affect cognitive function (e.g., head trauma, intracranial hemorrhage, traumatic brain injury, or intracranial malignancy), impaired vision or significant upper extremity weakness precluding use of a computer, inability to communicate in English or were unable to, or declined to provide informed consent. They were also excluded if they had been on hemodialysis for longer than 12 months.

Patients were screened for eligibility using electronic medical records as well as paper charts and dialysis records. Eligible patients were approached during hemodialysis and written informed consent was obtained prior to commencing the study. Demographic and clinical data were collected from electronic medical records and dialysis run sheets from the date of assessment (if the patient completed the tests during dialysis) or the dialysis run prior to the date of assessment (when the patient completed the assessment on the day following dialysis).

Patients completed cognitive assessment using a study tablet during hemodialysis or using a personal computer or tablet if the tests were completed at home after dialysis or on the day following dialysis. Investigators assisted patients by creating a personalized login and password on the CBS study webpage. As part of the CBS battery, there are standardized sets of written and pictorial instructions and a short instructional video preceding each test. Patients were provided with as much time as they needed to review the instructions prior to beginning each test. Each test was completed in sequence until the entire battery of 12 tests was finished or they were unable to continue due to testing-related fatigue.

### Statistical Analysis

Patients were defined as having cognitive impairment on a given test if their raw score was 1.5 standard deviations (SD) below age- and sex-matched controls derived from the CBS normative database (25). We then compared patients' cognitive performance with available data from healthy age and sex-matched control data by converting raw scores into *z*-scores. To determine patients' scores on each of the three cognitive domains (reasoning skills, short-term memory, and verbal processing), the z-score for each individual test was multiplied by a value that reflected the contribution of that test to each cognitive domain (i.e., factor loading) as established by Hampshire et al. (25). Patients' overall score on each cognitive domain was therefore the sum of the weighted (factor loaded) scores for that domain across all 12 tests. The resulting scores are designed such that the healthy population mean on each cognitive domain is 0 and the SD is 1.0. For patients who did not complete all 12 tests, we replaced missing scores with their expected values given the observed test scores and the known correlation structure among the tests in the population. The correlation structure between the 12 tests in the CBS battery was derived from a sample of 44,600 individuals (25). This conditional-mean replacement method has been shown to be most accurate when calculating principal component analysis scores in the presence of missing data (31). We then calculated a z-score for each patient on each of the three cognitive domains based on the abbreviated CBS and compared this score to the z-scores calculated based on the complete CBS battery.

Study patients were defined as having CI if they had a *z*-score < -1.5 in at least one of the three aforementioned cognitive domains. They were then stratified into two groups according to absence vs. presence of CI. Demographic and clinical variables were reported using descriptive statistics, expressed as frequency and percentage for categorical variables and mean ± SD for continuous variables. Comparisons between categorical and continuous variables were performed using Fisher's exact test and Mann-Whitney's *U*-test, respectively. An alpha < 0.05 was used as a cut-off to determine statistical significance.

## Results

**Figure 1:**
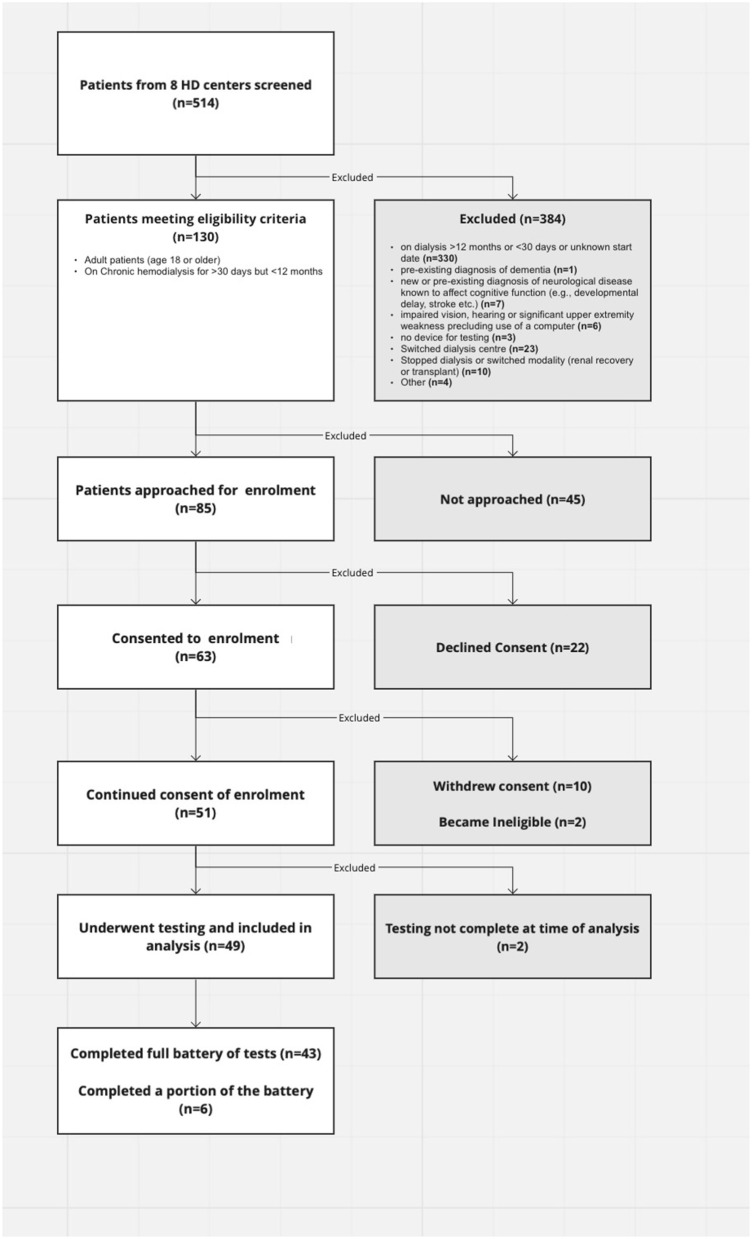
Flow chart of participant enrollment and testing.

Eighty-five of one-hundred thirty eligible patients who were within their first year of hemodialysis treatment in Southwestern Ontario were approached to participate in this study. We ultimately had 43 participants complete the full battery of tests and 6 complete part of it. Twenty-two declined participation and 10 withdrew from the study (Figure 1).

Baseline characteristics for the included patients and details of their hemodialysis are summarized in Table 1. The majority (35/49, 71%) of patients enrolled were from satellite dialysis units—units outside of major tertiary centers. The average age was sixty-three, 53% were female and all participants were caucasian. Approximately half had diabetes and the majority (95%) had hypertension. The most common etiology of renal disease was diabetes. The average dialysis vintage (duration of hemodialysis therapy prior to undergoing cognitive assessment) was 6 months (minimum 2 months; maximum 11 months). The average ultrafiltration rate (UFR) was 6.5 ml/kg/h although there was a wide range from 0.35 to 20 ml/kg/h.

**Table 1:** Patient demographic and clinical characteristics.

**Characteristic**	***n* (%), or mean (±SD)**
Female sex	26 (53%)
Age	62.6
**Comorbidities**	
Hypertension	46 (94%)
Diabetes	25 (51%)
TIA/stroke	8 (16%)
Mental health	8 (16%)
Sleep apnea	15 (31%)
HF	11 (22%)
CAD/IHD	22 (45%)
Atrial fibrillation	8 (16%)
Vascular disease	5 (10%)
**Etiology of renal disease**	
Diabetes	18 (37%)
Hypertension	7 (14%)
GN	7 (14%)
Obstructive/reflux/structural	6 (12%)
PCKD	2 (4%)
Drug or other	9 (18%)
**Dialysis details**	
HD vintage	6.1 months (±2.8) [mean (SD)]
Lowest systolic BP	117 (±19.9) [mean (SD)]
Average UF rate	5.85 ml/kg/h (±3.83) [mean (SD)]
Duration of treatment	211.2 min (±27.6) [mean (SD)]
Location of hemodialysis	14 (29%)—tertiary care center 35 (71%)—satellite unit
**Cognitive testing location**	
Testing completed at/during hemodialysis	19 (39%)
Testing completed at home	30 (61%)

Ten participants (20%) had issues completing the tests including fatigue, frustration and technological issues.

**Figure 2:**
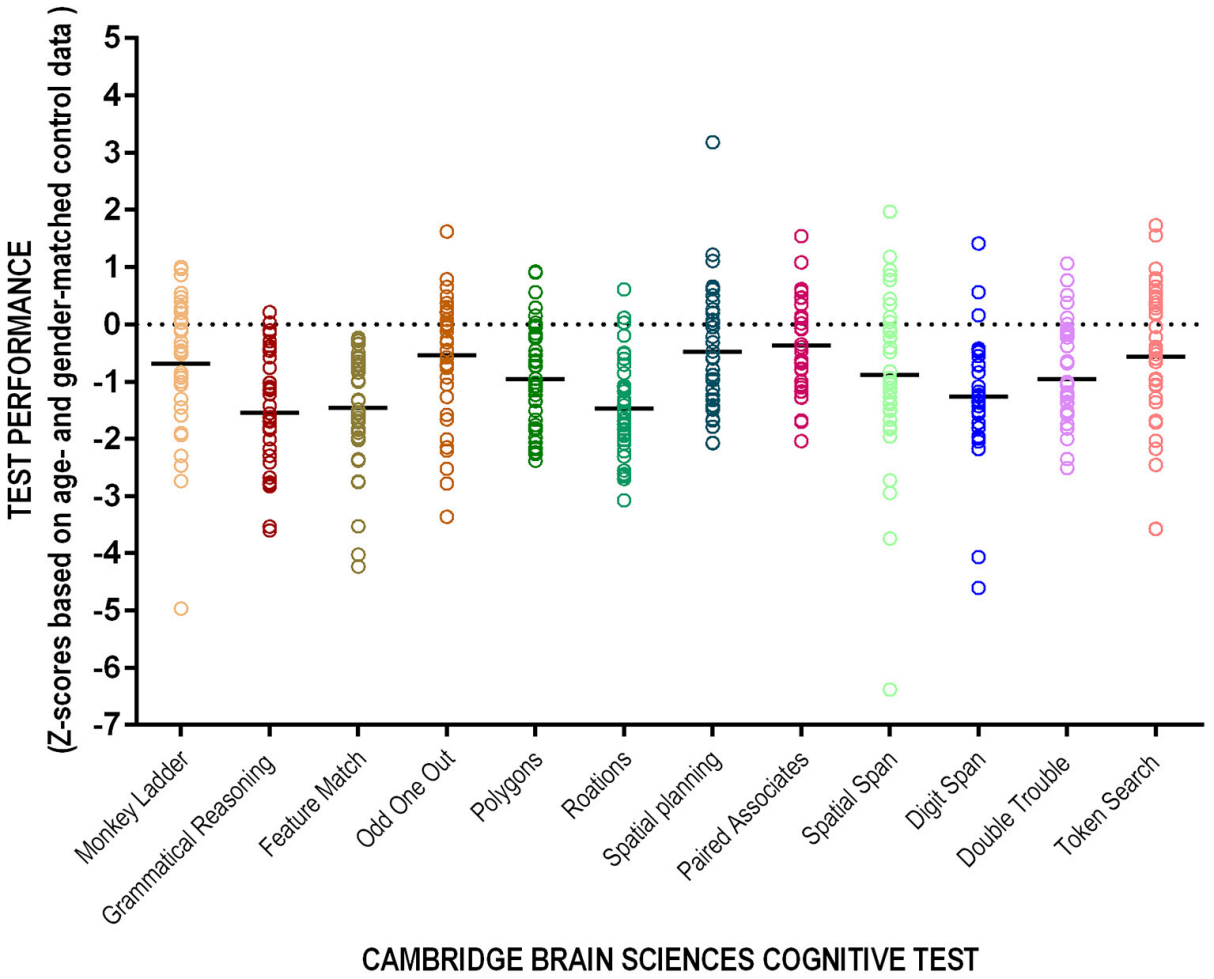
Performance on the 12-test CBS. Individual patient (circles) and cohort average (solid line) presented as *z*-scores corrected for age and sex.

Of the 43 patients who completed the full battery, nine (18%) did not meet criteria for cognitive impairment on any test; the average participant had scores qualifying for cognitive impairment in 3.7 tests (SD). The average scores for patients on dialysis were consistently below those of their age and sex-matched controls although only three tests had average z-scores consistent with cognitive impairment (Table 2). The majority (82%) of patients had cognitive impairment on at least one test and 35% had impairment on at least two (Table 3). Figure 2 shows individual participant as well as cohort average scores for each of the 12 tests.

**Table 2:** Cognitive performance of HD patients relative to age and sex-matched controls.

Cognitive test^domain tested^*^^/domain	**Mean *z*-score (SD)**	**Statistics (*t*-test, *p*-value)**
**CBS cognitive test**		
Feature match^a, b, c^	−1.47 (0.98)	*t*(47) = −10.28, *p* <0.0001
Odd one out^a, b^	−0.64 (1.17)	*t*(45) = −3.67, *p* = 0.0007
Polygons^b, c^	−1.02 (0.87)	*t*(47) = −8.04 *p* <0.0001
Rotations^b, c^	−1.58 (0.82)	*t*(45) = −12.93, *p* <0.0001
Spatial planning^a, b^	−0.52 (1.04)	*t*(45) = −3.35, *p* = 0.0016
Monkey ladder^a^	−0.52 (1.46)	*t*(47) = −2.36, *p* = 0.0227
Paired associates^a^	−0.55 (0.87)	*t*(45) = −4.24, *p* = 0.0001
Spatial span^a^	−0.86 (1.31)	*t*(47) = −4.50, *p* <0.0001
Token search^a^	−0.37 (1.01)	*t*(44) = −2.43, *p* = 0.0193
Digit span^c^	−1.20 (0.90)	*t*(43) = −8.74, *p* <0.0001
Double trouble^a, b, c^	−0.97 (0.84)	*t*(46) = −7.83, *p* <0.0001
Grammatical reasoning^b, c^	−1.57 (0.98)	*t*(45) = −10.75, *p* <0.0001
**Cognitive domain**		
Reasoning skills	−1.37 (1.16)	*t*(49) = −8.27, *p* <0.0001
Short-term memory	−0.41 (1.24)	*t*(49) = −2.32, *p* = 0.0250
Verbal processing	−1.47 (0.97)	*t*(49) = −10.61, *p* <0.0001

* *Each task can test more than one cognitive domain; the main cognitive domain(s) tested are noted here*.

a *Short term memory*.

b *Reasoning*.

c *Visual*.

**Table 3:** Participants with scores consistent with cognitive impairment in each domain and total number of domains.

***N*** **(%) with scores consistent with cognitive impairment**				
Reasoning skills	22 (45%)			
Short-term memory	10 (20%)			
Verbal processing	27 (55%)			
**Cognitive impairment across domains**				
	**None**	**One domain**	**Two domains**	**Three domains**
*N* (%)	9 (18%)	23 (47%)	15 (31%)	2 (4%)

**Figure 3:**
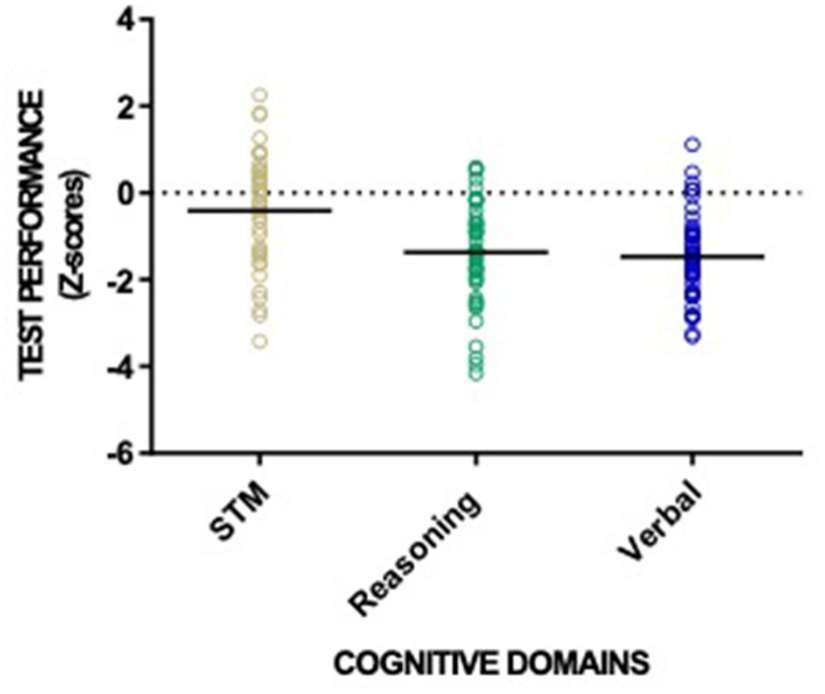
Patient performance categorized into the three CBS cognitive domains: reasoning skills, short-term memory and verbal processing. Individual patient (circles) and cohort (solid line) performance presented as z-scores with healthy normal mean = 0 and standard deviation =1.

Across all three cognitive domains, 23 (47%) patients qualified as having CI in one cognitive domain, 15 (31%) in two, 2 (4%) as having CI in all three (Table 3). Figure 3 shows individual test performance in each domain: as a whole, HD patients performed worse than healthy age and sex-matched controls. 9/49 patients (18%) qualified as having significant Short-Term Memory impairment (mean *z*-score −0.41 ± 1.24) compared to the normal population. In the domain of Reasoning, 21/49 (43%) of HD patients had *z*-scores showing significant impairment, (*z*-score −1.37 ± 1.16). HD patients had the poorest performance in Verbal domain with 27/49 (55%) meeting criteria for cognitive impairment (z-score −1.47 ± 0.97).

We investigated whether any differences in cognitive scores were evidenced after stratifying our study sample by major comorbidities (diabetes, hypertension, cerebrovascular disease, heart failure, ischaemic heart disease, atrial fibrillation, vascular disease, mental health conditions, and obstructive sleep apnea), but no significant differences were found (data not shown). Minor and non-statistically significant differences were evident after stratifying for hypertension, however, the groups were significantly unbalanced with 46/49 patients having hypertension. Similarly, no significant correlations between dialysis parameters (HD vintage, UF rate, lowest systolic blood pressure and time on dialysis) were detected in this population all characterized by having been established on dialysis for only a relatively short period (data not shown).

## Discussion

The results of our study show a high prevalence of CI within months of starting HD. Nearly half of our participants exhibited scores qualifying for CI in one domain, and a third with scores consistent with CI in two domains. CBS appears to be a meaningful and easily administered form of comprehensive cognition testing.

The majority of patients in our study were from satellite dialysis units; these patients are generally healthier and more independent than those being treated in tertiary care centers. The degree of CI that was seen in our results likely underestimates its prevalence in the general in-center HD population. This is supported by previous literature showing that CI affects in-center hemodialysis patients more so than other dialysis populations (13).

The pattern of CI observed in this study showed that reasoning and verbal processing skills were less preserved compared with short-term memory. To clarify, “reasoning skills” included visuospatial processing, deductive reasoning, and planning. Significant CI in the reasoning and verbal domains were seen in 43%, and 55% of participants respectively, compared with 18% showing impaired short-term memory. These results are partially in line with previous findings. Murray et al. explored cognitive performance (memory, executive function, language) in a sample of 338 HD patients with standard neuropsychological testing: although only 2.9% had a known history of CI, the authors found that only 12.7% patients did not exhibit any CI. Furthermore, the authors found that memory and executive function were impaired in 35–41% cases, respectively, as opposed to 11% having impaired verbal domain (4). This might reflect lower dialysis vintages; in a study where dialysis vintages were higher (median 57 months), memory and language were most severely impaired in those with mild cognitive impairment and attention and visuospatial functions were more severely impaired in those with severe cognitive impairment. Further, this study showed a correlation between higher dialysis vintage and major cognitive impairment (32). Although results from this study are challenging to compare due to different cognitive testing methodologies, both studies show significant impairment in executive function, which is typically associated with cerebral small vessel disease and leukoaraiosis: indeed, executive function relies on deep white matter connectivity. In support of this finding, brain diffusion tensor imaging magnetic resonance has shown evidence of white matter ischemic injury associated with intradialytic hemodynamic instability (33). Another study of older Chinese HD patients and found a high frequency of CI with attention and visuospatial domains the most impaired. This was a larger study including over 600 participants and found age, education level, history of stroke, hypertension, dialysis vintage and single-pool Kt/V to be contributing factors to CI (32). Another risk factor is reduced cerebral venous oxygenation which has been demonstrated to occur in HD patients and correlated with reduced performance on cognitive testing (12). A second study also demonstrated reduced deep regional cerebral venous oxygenation in HD patients as well as reduced cognition although the two did not correlate. This may be a result of different testing approaches as the first study used the MoCA and the second used the mini mental status exam (MMSE) which has been shown to be a less effective test of cognition in this population (24).

To the best of our knowledge, this was the first use of the CBS in hemodialysis patients. While many validated measures of cognitive function have been used in the CKD and ESRD populations (24, 34), most tests require the time and presence of trained personnel for administration and scoring and require in-person testing. These human resources are not routinely available in tertiary care centers, much less in the satellite dialysis units. Further, patients on dialysis are often limited in terms of mobility, fatigued by their dialysis treatment and rely on pre-booked transportation. This means staying late or returning to a hospital setting for testing is difficult; treatment-related fatigue may limit ability and willingness to complete tests. In this study, although some participants had difficulties, the majority had no problem completing the tests. Toward the end of our study, based on our participant feedback, the CBS was able to adjust their testing to allow patients to save their progress and resume testing within a 72-h period. The timing of testing is an important consideration, although the current evidence remains ambiguous. One study suggests improvement in CI as quickly as 1 h post HD, and better cognition both a day prior and following HD treatment (35). Another study demonstrated decreased evoked potential latency for 24 h post HD treatment with progressive increase thereafter; it also demonstrated best performance on CI testing at 24 h post treatment (36). In a third study, HD and control participants underwent cognitive testing twice (the day prior to and immediately following dialysis for the HD patients). This study found increased CI in the HD group compared to controls however, on the individual level, they found stable performance on cognitive testing overtime (37). Of the patients who completed testing in our study, 27 (>60%) did so in the interdialytic period. Considering most participants in our study tested during the interdialytic period and still demonstrated CI, this may be under-representing the degree of CI that occurs during the HD period. This may, however, be a more relevant time to assess as patients spend more of their time in the interdialytic period compared to time on HD therapy. An issue with the current evidence is that it is all based on small sample sizes. The question of when the best time to test for cognitive function in HD is important and needs answering based on a large study that measures cognitive performance over multiple time points.

Additionally, as the CBS compares study scores to matched controls using z-scores, we are better able to attribute CI to hemodialysis rather than to age-related risk. The CBS is therefore a potentially promising tool to screen for, diagnose and monitor CI in this patient population. The web-based platform and automated scoring allows for inclusion of patients who otherwise might not have the opportunity for in-person cognitive assessment and follow-up.

This study has several limitations. This was a cross-sectional study and does not allow attribution of causality based on any observed relationships. A few of the included patients had comorbidities that may impact CI including history of TIA or stroke, obstructive sleep apnea (OSA) and mental health disorders such as anxiety and depression. However, these are also common comorbidities in the HD population that should be taken into consideration when assessing cognition - HD therapy may be a contributing factor to these comorbid conditions. In a cross-sectional study in Saudi Arabia, nearly 20% of HD patients had anxiety at 25% depression (38). In another study, nearly 85% of hypertensive HD patients had depression (39). The prevalence of anxiety has been estimated at up to 52% in HD patients (40). Sleep apnea is also far more prevalent in the HD population than the general population with multiple studies suggesting a prevalence of >50% (41–43). The high prevalence of these conditions is important and may contribute to CI in this population and therefore, patients with these comorbidities should be included in assessments of cognition. While our sample size limits our ability to assess the impact of these on cognitive performance, a larger scale study including patients with these conditions could better elucidate the role they each play in cognitive decline.

Additionally, participants were matched based on age and sex to a control group; matching did not take into consideration other comorbidities or match based on underlying disease without the need for dialysis. When stratified by comorbidities and dialysis parameters, however, we did not find any signal of correlation. The study was not powered for this analysis and the effects of these factors on CI may be trivial. This suggests that there might be factors intrinsic to HD that are more relevant to the development of CI. Intradialytic hypotension is a likely mechanism associated with CI in HD patients (13, 44). indeed, cumulative exposure to intradialytic hypotension has been linked to new-onset dementia in HD patients (45). From a pathophysiological standpoint, ultrafiltration rates and intradialytic hypotension are associated with cerebral hypoperfusion and white matter injury (46–48). Intradialytic cerebral blood flow decline has correlated with ultrafiltration volumes and a measurable decline in executive function. This persists in those who remained on dialysis but not those who undergo renal transplant (49, 50). Compared to patients on PD, HD patients have worse cognitive function and higher rates of dementia (15). HD patients have been shown to have higher prevalence of brain atrophy than the general population and that loss of gray matter is seen more rapidly in dialysis patients than CKD and is associated with loss of executive function (51). One study found lower cerebral venous oxygen saturation in patients on HD compared to healthy controls and cognitive function was also statistically significantly lower in the HD group (12). Timing of neuropsychological testing (intradialytic vs. non-dialysis day) has been shown to affect cognitive performance in HD patients. Cognitive performance has been suggested to be optimal 24 h after HD, whereas it is likely negatively affected by intradialytic osmolar shifts and cerebral hypoperfusion (13, 52). In homogeneous conditions for CBS testing in this study may have led to overestimations of CI in our study sample, especially in the verbal skills domain. A future, large-scale study, using patients as their own controls and measuring CI using the CBS longitudinally in the CKD phases, and over time during chronic HD and even after transplantation would be helpful in determining the direct effects of uremia, HD and duration of HD on CI.

In conclusion, this study demonstrates that CBS testing provides an effective way to readily screen for CI in the maintenance HD population. We showed evidence of CI early after initiating HD with a more pronounced effect on reasoning and verbal processing skills. The CBS battery would be an effective tool in larger scale studies to examine changes in cognition over time, to explore the correlation between CBS with recognized risk factors for CI associated with HD, and to test interventions aimed at preventing or slowing the progression of CI in the HD population.

## Data Availability Statement

The raw data supporting the conclusions of this article, if requested, will be made available by the authors without undue reservation

## Ethics Statement

The studies involving human participants were reviewed and approved by Western University Research Ethics Board, study identification number 111721. The patients/participants provided their written informed consent to participate in this study.

## Author Contributions

CM conceived, designed, and supervised the study. MZ and CH recruited and enrolled participants and collected data. MS, JG, CW, and FS synthesized and analyzed data. MS wrote and revised the manuscript with input and edits from FS and CM. All authors contributed to the article and approved the submitted version.

## Funding

CM is the recipient of a funding award from Western University's BrainsCAN - a neuroscience research initiative. MS was the recipient of the 2020 PSI Research Trainee Fellowship which provides salary support for researching training.

## Conflict of Interest

The authors declare that the research was conducted in the absence of any commercial or financial relationships that could be construed as a potential conflict of interest.

## Publisher's Note

All claims expressed in this article are solely those of the authors and do not necessarily represent those of their affiliated organizations, or those of the publisher, the editors and the reviewers. Any product that may be evaluated in this article, or claim that may be made by its manufacturer, is not guaranteed or endorsed by the publisher.
